# The epidemiology and natural history of depressive disorders in Hong Kong's primary care

**DOI:** 10.1186/1471-2296-12-129

**Published:** 2011-11-24

**Authors:** Weng Yee Chin, Cindy LK Lam, Samuel YS Wong, Yvonne YC Lo, Daniel YT Fong, Tai Pong Lam, Peter WH Lee, Josephine WS Wong, Billy CF Chiu, Kit TY Chan

**Affiliations:** 1 Department of Family Medicine and Primary Care, the University of Hong Kong, 3/F Ap Lei Chau Clinic, 161 Main Street, Ap Lei Chau, Hong Kong; 2 School of Public Health and Primary Care, Chinese University of Hong Kong, Hong Kong; 3 School of Nursing, the University of Hong Kong, 4/F, William M.W. Mong Block 21 Sassoon Road, Pokfulam, Hong Kong; 4 Department of Psychiatry, the University of Hong Kong, Queen Mary Hospital, 102 Pokfulam Road, Pokfulam, Hong Kong; 5 Hong Kong Sanatorium and Hospital, 2 Village Road, Happy Valley, Hong Kong; 6 In private practice

## Abstract

**Background:**

Depressive disorders are commonly managed in primary care and family physicians are ideally placed to serve as central providers to these patients. Around the world, the prevalence of depressive disorders in patients presenting to primary care is between 10-20%, of which around 50% remain undiagnosed. In Hong Kong, many barriers exist preventing the optimal treatment and management of patients with depressive disorders. The pathways of care, the long term outcomes and the factors affecting prognosis of these patients requires closer examination.

**Methods/Design:**

The aim of this study is to examine the prevalence, incidence and natural history of depressive disorders in primary care and the factors influencing diagnosis, management and outcomes using a cross-sectional study followed by a longitudinal cohort study.

Doctors working in primary care settings across Hong Kong have been invited to participate in this study. On one day each month over twelve months, patients in the doctor's waiting room are invited to complete a questionnaire containing items on socio-demography, co-morbidity, family history, previous doctor-diagnosed mental illness, recent mental and other health care utilization, symptoms of depression and health-related quality of life. Following the consultation, the doctors provide information regarding presenting problem, whether they think the patient has depression, and if so, whether the diagnosis is new or old, and the duration of the depressive illness if not a new diagnosis. If the doctor detects a depressive disorder, they are asked to provide information regarding patient management. Patients who consent are followed up by telephone at 2, 12, 26 and 52 weeks.

**Discussion:**

The study will provide information regarding cross-sectional prevalence, 12 month incidence, remission rate, outcomes and factors affecting outcomes of patients with depressive disorders in primary care. The epidemiology, outcomes, pathways of care, predictors for prognosis and service needs for primary care patients with depressive disorders will be described and recommendations made for policy and service planning.

## Background

Depression is a common and serious disorder that results in decline in functioning and impairment to quality of life. Unfortunately, despite the availability of effective treatments, many barriers exist preventing the optimal detection and management of these patients. Depression has been identified as a major public health issue in many parts of the world including Hong Kong [1]. In Hong Kong, the 12-month general population prevalence of depressive disorders among adults aged 18-65 years has been estimated to be approximately 8.4% [2]. This is similar to the rates in Canada (8.2%) and the US (8.7%) [3] but higher than those in Australia (6%) [4]. The rate of doctor-reported depressive disorder in Hong Kong however has been recorded as only 1.5% [5]. This discrepancy implies that there must be a large number of people in our community with undiagnosed depression.

Around the world, the prevalence of depressive disorders in patients presenting to primary care has been estimated to be between 10-20%, of which around 50% remain undetected by doctors [6]. The primary care setting is the point of entry for most people into the health system and primary care clinicians are ideally placed to serve as the central health care provider for patients with depressive disorders. The choice and effectiveness of intervention by primary care doctors can have a profound effect on the quality of life of the patients and the demand for services. However, many challenges exist in providing optimal care, including difficulties in recognizing patients with depression, developing an adequate diagnostic initial assessment, implementing treatment and management strategies, and integrating care of depression with that of co-existing chronic illnesses [7].

Depressive disorders are regarded as high prevalence, chronic conditions with significant impact on quality of life. Nevertheless, meta-analyses of the long-term outcomes and factors affecting prognosis remain far from clear [8]. Studies have shown that one-third to one-half of primary care patients with depressive disorders have symptoms that persist over six to twelve months, and that severity of symptoms, age and presence of co-morbidity were predictors of this persistence [6,9]. Unfortunately, little else is well understood and much controversy still exists regarding the significance of detection of these patients on prognosis and clinical outcomes over time [10]. This lack of clarity may in part be due to the absence of a 'gold standard' by which primary care doctors diagnose depression, and in part to the complexity by which multiple factors are considered, before deciding whether or not to label a patient or initiate therapy [11]. More recent studies are revealing a more realistic picture, that diagnosis and management decisions are often not made on initial consultation, but occurs gradually over a period of longitudinal care [12].

In order to make recommendations regarding mental health policy and the planning of mental health services, it is necessary to have current knowledge of the epidemiology and outcomes of depressive disorders managed in the primary care setting. At this point, there is little local data available regarding the prevalence and incidence of depression in Hong Kong's primary care settings and no similar studies examining the longitudinal outcomes and prognosis of these patients.

## Methods/Design

### Study Design and Objectives

We aim to examine the epidemiology and naturalistic history (i.e. outcomes of usual care with no added intervention) of depressive disorders in Hong Kong's primary care using a cross-sectional study followed by a longitudinal cohort study with the following objectives:

#### Cross-sectional study

1. Estimate the cross-sectional prevalence of depression in primary care and its associated demography and co-morbidities;

2. To examine the criteria by which primary care doctors diagnose depression and the factors that influence this decision;

3. To describe how primary care doctors manage depression and the factors influencing their treatment choices.

#### Longitudinal Cohort Study

1. To study the natural history of depression and factors that can predict remission;

2. To estimate the 12 month incidence of depression in a primary care patient population;

3. To examine the patients' pathways of care and the factors influencing treatment.

### Participants and Sampling

#### Cross Sectional Study(Figure 1)

Doctors working in primary care practices across Hong Kong have been invited to participate in this study. The doctors were recruited from the mailing list of the Hong Kong College of Family Physicians (HKCFP) and are comprised of clinicians working in private practice, the public sector and non-profit, non-governmental organizations.

**Figure 1:**
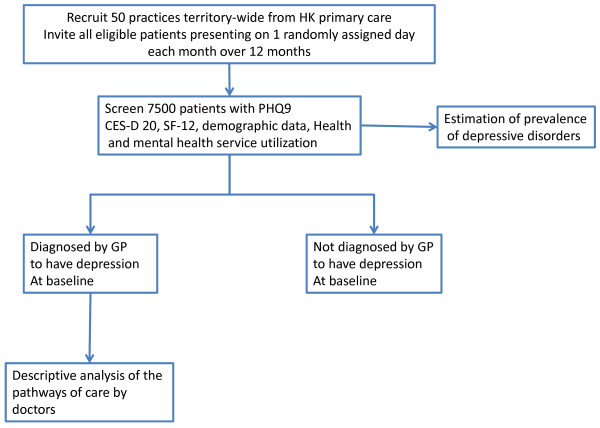
**Cross-sectional study design**.

All eligible patients presenting to the study doctor on one randomly selected day each month during the study period are invited to participate. The recruitment days are randomly generated but exclude Sunday and public holidays (when most primary care clinics are closed). The twelve month baseline collection period attempts to account for seasonal variability and to improve representation of the primary care case load. Patients are excluded if they are < 18 years, cannot speak English, Cantonese or Mandarin, have cognitive or communication difficulties, have previously been recruited, or are not consulting the doctor (e.g. vaccination, wound care or prescription refill).

A coded, anonymous questionnaire containing consent form, questions regarding ethnicity, demographic details, household income, co-morbid medical conditions, family history of mental illness, previous doctor-diagnosed mental illness, recent mental and other health care service use, as well as the PHQ-9, the CES-D 20 and the SF-12v2 is self administered by each patient whilst in the waiting room. The questionnaires are available in both English and Chinese. A trained, bi-lingual research assistant is present to explain the study, obtain consent, and distribute and collect questionnaires. If the subject is unable to self-administer the questionnaire due to poor literacy, the research assistant helps to administer the questionnaire.

The doctors are asked to complete a clinical data collection form for each recruited patient, recording the presenting problem, their opinion on whether the patient has depression, and whether the diagnosis is new or old. If the doctor reports a diagnosis of depression, further data is collected regarding the duration of illness, date of initial diagnosis and management.

#### Longitudinal Study (Figure 2)

As part of the baseline cross-sectional survey, patients are invited to participate in a 12 month cohort study to evaluate the incidence and naturalistic outcomes of care. Those who consent to enter the cohort study by providing their names and contact numbers are subsequently followed up by telephone (by an interviewer who is blinded to the initial screening results) with a modified version of the initial questionnaire and repeated at 2 (for PHQ-9 positive subjects), 12, 26 and 52 weeks. The participants of the longitudinal study are stratified according to their initial PHQ-9 screening result:

**Figure 2:**
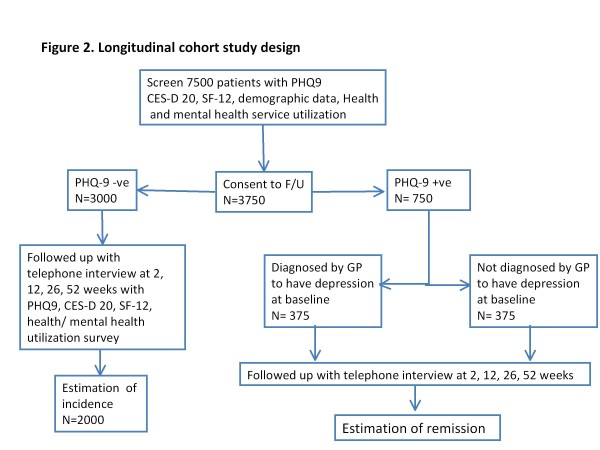
**Longitudinal cohort study design**.

Those who are screened negative for depression at baseline (PHQ-9 score < 9), are followed up to determine the incidence of depression, and as a control group for the changes in quality of life.

Those who are screened positive (PHQ-9 score≥9) for depression are divided into two groups: those where the doctor has recorded a diagnosis of depression, and those where the doctor has not recorded a diagnosis of depression.

The two groups are followed to examine for changes in symptom severity PHQ-9, quality of life, symptom remission and health care utilization over the 12 months and for comparison between the two groups. Symptom remission is defined as the absence of significant depressive symptoms defined by PHQ-9 score < 5 together with CES-D 20 score < 16.

### Study instruments and screening tools

**A. The Patient Health Questionnaire 9 (PHQ-9)**. The PHQ-9 is a self-report depression component of the Primary Care Evaluation of Mental Disorder Procedure (PRIME-MD) [13] which has been validated in primary care for diagnosis of depression. It scores each of the 9 DSM-IV criteria from 0 (not at all) to 3 (nearly every day). It has been calibrated against the Chinese Hamilton Depression Scale (CHDS) for screening use in Hong Kong primary care. Using a cut-off point of 9, this tool has a sensitivity of 80% and specificity of 92% for the diagnosis of depression [14]. The PHQ-9 can also be used to measure severity of symptoms (score 1-4 minimal, 5-10 mild, 10-14 moderate, 15-19 moderately severe, 20-27 severe) and can be used for monitoring symptom progression or remission over time [15].

**B. The Centre for Epidemiologic Studies Depression Scale (CES-D 20)**. The CES-D 20 is a widely used self-report scale which measures the current level of depressive symptomatology in the general population, with an emphasis on depressed mood during the past week [16]. It has been validated in community and primary care populations, and has good test-retest reliability [17]. It includes 20 items reflecting major dimensions of depression: depressed mood, feelings of guilt and worthlessness, feelings of helplessness, psychomotor retardation, loss of appetite, and sleep disturbance. It has been translated for use in Hong Kong adults and has been used in epidemiological studies of the Hong Kong general population [5,18]. Scores range from 0 to 60, with higher scores indicating more severe symptoms of depression. CES-D 20 scores of 16 to 26 are considered indicative of mild depression and scores of 27 or more indicative of major depression.

**C. The SF-12 Health Survey Version 2.0 (SF-12 v2)**. The SF-12 v2 is a 12-item abbreviated version of the SF-36^® ^Health Survey that assesses health-related quality of life. The SF-12v2 generates two norm-based summary scores; a mental component score and a physical component score. In a general population the mean score on each component is 50. The SF-12v2 has been translated and validated for use in the Hong Kong primary care setting [19].

**D. Personal data questions **on socio-demography and co-morbidity is adapted from previously primary care patient surveys performed in Hong Kong [20].

### Sample size calculation

For the estimation of the prevalence of depression by PHQ-9, we estimated the sample size to ensure an error of < 2% for an anticipated prevalence of 20% [21]. Without the consideration of the design effect due to cluster sampling by practice, we need 1540 subjects in total by a 95% confidence interval. The intraclass correlation (ICC) for the intra-cluster correlation was taken as 0.02 which is slightly conservative to the one reported in the DIAMOND study [21]. With 50 practices and after accounting the design effect due to cluster sampling, 80 subjects per practice are needed.

For the comparison of the two component scores of the SF-12v2 between subjects diagnosed with depression and are PHQ-9 positive and subjects with undiagnosed depression and are PHQ-9 positive, the sample size calculation was based on the analysis by mixed effects model taking account of the clustering effect by practice. To adjust multiplicity for the comparisons of the two component scores, a 2.5% nominal level of significance was used (by Bonferroni adjustment) in order to control the overall false positive error rate below 5%. In order to have 80% power to detect an effect size of 5 with a slightly conservatively estimated standard deviation of 15 [19] and assume small within cluster correlation of 0.02, 10 subjects per practice for 50 practices are needed [22]. Assuming 50% of the subjects who are initially screened consent to participate in the longitudinal study and 20% of them are PHQ-9 positive with a 30% attrition rate, 150 subjects per practice are needed.

For the estimation of the 12-month incidence of depression on subjects who have no depression by PHQ-9 at baseline and consent to participate in the longitudinal follow-up study, the sample size was calculated to ensure an error within 1% for a conservatively estimated 12-month incidence of depression of 3% (incidence of depression was 1% to 3% in the literature) [23]. The ICC for the intra-cluster correlation was again taken as 0.02. With 50 practices and taking into account of the design effect due to cluster sampling, 40 subjects without depression per practice are needed who are followed for 12 months by a 95% confidence interval [24]. Assuming 50% of the subjects who are initially screened consent to participate in the longitudinal study, 80% of them have no depression in initial screening, and a 30% attrition rate, 150 subjects per practice are needed.

For the estimation of the resolution rate at 12 months in subjects who are PHQ-9 positive at baseline and consent to participate in the longitudinal follow-up study, the sample size was calculated to ensure at most 5% error in a 95% confidence interval for a conservatively estimated 12-month resolution rate of depression of 30% [9]. The ICC for the intra-cluster correlation was again taken as 0.02. With 50 practices and taking into account the design effect due to cluster sampling, 8 subjects per practice who are followed for 12 months are needed [24]. Assuming 50% of the subjects who are initially screened consent to participate in the longitudinal study, 20% of them have depression in initial screening, and 30% attrition rate, 115 subjects per practice are needed.

As a result, 150 subjects per practice are needed for a total of 7500 subjects from 50 practices.

### Outcome Measures

The primary outcomes of this study are:

1. the cross-sectional prevalence of depressive disorders in primary care

2. the 12-month incidence rate of depressive disorders in a primary care population

3. the quality of life of patients with depressive disorders over one year

4. the remission rate over one year

The secondary outcomes of this study are:

1. how patients with depressive disorders are being managed in primary care

2. factors influencing the clinical diagnosis of depression

3. factors affecting the outcomes of depressive disorder

4. the predictors of prognosis of depressive disorders

Possible factors affecting the above outcome measures will be categorized into:

a) Patient factors: socio-economic status, demography, smoking, drinking, co-morbidity, family history and illness severity

b) Doctor factors: socio-demography, previous education and training, system setting (private vs. public) and access to service

### Statistical analysis

#### Cross-sectional analysis

The estimation of the prevalence of depression will be made on the subjects screened at baseline by a 95% confidence interval. The clustering by practice will be taken into account [25]. In addition, the influence of patient and doctor factors on depression will also be explored by a non-linear mixed effects model with logit link that takes account of the within practice correlation. Specifically, patient factors will be considered as level 1 covariates and doctor factors will be considered as level 2 covariates. Similar analyses will be pursued on the diagnosis of depression by doctors.

#### Longitudinal analysis

The 12-month incidence of depression among the PHQ-9 negative group at baseline will be similarly estimated as in the prevalence of depression. Patient and doctor factors associated with the incidence of depression will also be examined by non-linear mixed effects model. The physical and mental component scores of the SF-12v2 as well as other health outcomes of PHQ-9 positive subjects will be compared by a linear mixed effects model. Confounding factors, including socio-demographics will be adjusted. Effects of patient and doctor factors will also be examined in a linear mixed effect model analysis with patient factors taken as level 1 covariates and doctor factors taken as level 2 covariates. The same analyses will be performed for the 12-month resolution rate of depression in subjects who are PHQ-9 positive at baseline.

A 5% level of significance will be used and all estimates will be accompanied by a 95% confidence interval where appropriate. The Statistical Analysis System (SAS) or other competent statistical software will be used to carry out the analyses.

### Ethics Approvals

This study has received ethics approval by the Institutional Review Board of the University of Hong Kong/Hospital Authority Hong Kong West Cluster, the Research Committee of Hong Kong Sanatorium and Hospital, the Research Ethics Committee for Hong Kong Hospital Authority Kowloon East and Kowloon Central, and the Joint Chinese University of Hong Kong and Hong Kong Hospital Authority New Territories East Clinical Ethics Review Committee.

## Discussion

The establishment of a primary care practice-based study involving a large number of individual practices requires a great deal of preliminary coordination, and there were a number of initial challenges which had to be overcome.

### Recruitment of Doctors and Practices

As there is no comprehensive registry of primary care providers in Hong Kong, we used the 1500-member mailing list of the Hong Kong College of Family Physicians (HKCFP) as our sampling population from which we invited doctors to participate. Following the initial mailing and follow-up bulk-email, we received only 70 responses. Of those who responded, three were retired or no longer practicing in Hong Kong, 12 were not eligible (employer did not allow their participation in the study or doctor did not work in a primary care setting), and 15 declined after receiving more information regarding the study. For the 15 who declined, their cited reasons were concerns regarding the impact of the study on their tight working schedule, that they saw too few patients, and concerns regarding patient confidentiality. Of the 40 doctors that were eligible, the demographic spread was unevenly distributed. To overcome this, we employed a snow-balling technique (asking doctors to refer local colleagues to the study) in under sampled demographic areas which proved to be very useful. By the end of the doctor recruitment period we had 60 doctors from 45 different practice locations demographically distributed across Hong Kong. In terms of type of practice, 25 of the 60 doctors worked in private solo clinics, 8 in private group clinics, 11 in private hospital clinics, 11 in public-sector clinics, 2 in university health services, and 3 in non-profit non-governmental organisations.

### Patient recruitment

Although a standardized patient recruitment procedure was initially developed, recruitment logistics had to be tailor-made for each practice site due to significant variability in patient flow and logistics. Patient recruitment has been quite straight forward at solo private practices, but we have encountered difficulties at large multi-disciplinary clinics due to high patient volumes coupled with shorter consultation times. At these large clinics, identifying which patients to approach has been a major obstacle to subject recruitment. This barrier can be overcome by enlisting the help of nursing and clerical staff to identify appropriate patients, and by sending two field workers per doctor to minimise missed patients.

### Study Instruments

At baseline and all follow-up interviews, both the PHQ-9 and CES-D 20 are administered. We found that some patients (particularly the elderly) got annoyed as it seems that the same question is asked twice, and some items are difficult for them to answer. This can be overcome by reinforcing the training of the interviewers on how to best explain the items requiring clarification, and how to encourage the subjects to choose the best available answer even though they may not be absolutely sure.

### Response rate

Patient recruitment commenced over the various sites between October 2010 and April 2011. Response rates to the baseline questionnaire have ranged from 73.9% to 85.6%; however the response rate for the cohort study was initially as low as 25%. In an attempt to enhance the cohort response rate, we send quarterly newsletters to all our study doctors providing them with an update of the study progress, and encouraging them to promote the cohort study. We reduced the length of the follow-up questionnaires by removing redundant questionnaire regarding demography and health care preferences. We also modified the protocol by removing the 2-week telephone follow-up for those who were PHQ-9 negative at baseline. A very useful strategy has been to reinforce the training of our field workers on how to explain the study's purpose to the subjects, emphasising why the knowledge gained could benefit others. Following the implementation of these strategies, the cohort response rate rose to 48%.

### Staffing issues

Observational practice-based studies are logistically complex and can be manpower intensive. Maintaining a stable and well-trained team of research assistants is needed to enhance the efficacy and quality of practice-based studies such as ours. In total, it has been necessary to employ three full-time and three part-time research assistants to maintain patient recruitment. In addition, a casual staff pool has been used to conduct telephone follow-up interviews and input data. An experienced project coordinator to liaise between various parties, and who is available for urgent troubleshooting is essential for the smooth running of the study.

### Strengths and Weaknesses

One of the major strengths of this study has been our success in recruiting a large number of primary health care providers to collaborate in this study. There are many service delivery options for patients seeking primary care in Hong Kong and our wide sampling of practice types captures this diversity. To date, there has been no similar wide scale epidemiological study on the prevalence and incidence of depression in the Hong Kong adult primary care population. An unexpected side benefit has been the generation of interest in participating in research, and identification of a number of individual family physicians seeking further research skills development and academic collaboration.

As there is no comprehensive registry of primary care providers for Hong Kong, the mailing list of the Hong Kong College of Family Physicians was chosen as the primary sample frame for the doctors. A limitation of this study is that doctors who provide primary care, but are not members of the College, have not been deliberately sampled.

Recruitment for the cross-sectional study will continue until January 2012, and the cohort study until Jan 2013. The findings from this study should help provide information on the predictors of prognosis in primary care depression as well as identify which services are in greatest need and for whom they are most needed. If confirmed, these predictors can be incorporated into mental health policies to better help those at high risk of poor outcome, and enable decision-makers to more effectively allocate mental health resources for the community.

## Abbreviations

**CES-D 20: **Centre for Epidemiologic Studies-Depression Scale; **DSM-IV: **Diagnostic and Statistical Manual of Mental Disorders 4^th ^Edition; **HKCFP: **Hong Kong College of Family Physicians; **ICC: **Intra-class correlation; **PHQ-9: **Patient Health Questionnaire 9; **PRIME-MD: **Primary Care Evaluation of Mental Disorder Procedure; **SF-12v2: **SF-12 Health Survey Version 2.0; **CHDS: **Chinese Hamilton Depression Scale; **SCID: **Structured Clinical Interview for Depression

## Competing interests

The authors declare that they have no competing interests.

## Authors' contributions

CL initially conceived the study. All authors collectively designed and drafted the study protocol and sought funding and ethical approving. DF led on statistical analyses. CL, TPL, YL, BC, SW contributed to recruitment and data collection. PL and JW provided assistance with drafting and management of the suicide management protocol. KC was the project coordinator, recruited and trained the fieldworkers, assisted with recruitment of study doctors, coordinated the data collection, and contributed to the drafting of the manuscript. WYC is PI of the funding application, coordinated the research network and research team, and drafted the manuscript. All authors have read the draft critically and approved the final manuscript.

## Pre-publication history

The pre-publication history for this paper can be accessed here:

http://www.biomedcentral.com/1471-2296/12/129/prepub
